# The Safety and Complication Rate of Deep Plane Facelift Under Local Anesthesia

**DOI:** 10.1093/asjof/ojag156

**Published:** 2026-08-21

**Authors:** Sarah Novis

## Abstract

**Background:**

The deep plane facelift is traditionally performed under general anesthesia or intravenous (IV) sedation. Because of demand increases among older patients and those with comorbidities, interest has grown in performing comprehensive facelift surgery under local anesthesia; however, safety data remain limited.

**Objectives:**

The objective of this study is to evaluate the complication rate associated with deep plane facelift surgery performed under local anesthesia and to compare these outcomes with established complication rates reported in the literature.

**Methods:**

A retrospective review was conducted of 142 consecutive patients who underwent primary deep plane facelift and necklift under local anesthesia with oral sedation. All procedures were performed by a single surgeon in an office-based facility with a minimum follow-up of 90 days. Major and minor complications were recorded and compared with established complication rates for deep plane facelift reported in the literature using odds ratio analysis.

**Results:**

The mean patient age was 63.5 years. There were no major hematomas, skin necrosis, or temporary or permanent facial nerve injuries. Minor complications included seroma (3.52%), minor hematoma (0.70%), and localized infection (1.40%), all resolving with conservative management. No patients required hospitalization or experienced thromboembolic events. Complication rates did not differ significantly from published data (*P* > .05).

**Conclusions:**

Deep plane facelift with necklift can be safely performed under local anesthesia with oral sedation, demonstrating complication rates in this cohort that are no higher than published benchmark rates for patients receiving general anesthesia or IV sedation.

**Level of Evidence: 4 (Therapeutic):**

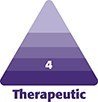

The demand for rhytidectomy surgery has increased in recent years, driven in part by the widespread adoption of the deep plane approach.^1,2^ This technique has set new expectations for natural and long-lasting outcomes.^3,4^ However, the deep plane approach is also a more extensive procedure that is most often performed under general anesthesia or intravenous (IV) sedation. An aging population has further amplified the demand for facial rejuvenation procedures. Because patients live longer, rhytidectomy is increasingly being requested in older individuals and in those with more medical comorbidities. Many of these patients express a preference for safer alternatives to general anesthesia and IV sedation, citing concerns about anesthetic exposure, particularly in the elderly.^5^

Facelift surgery under local anesthesia offers the potential to broaden patient eligibility and accessibility. Historically, local anesthesia has been employed in facelift procedures, although its use has largely been restricted to limited dissections or “mini-lifts.”^6-8^ Additionally, there have been concerns about the safety of performing facelift surgery under local anesthesia. One of the largest series examining facelifts performed under local anesthesia by Frojo et al reported an elevated complication risk, with 13.2% of patients developing hematomas.^9^ There have also been concerns about local anesthetic dosing and the risk of lidocaine toxicity in facelift procedures performed under local anesthesia.^10^ Studies on the safety of deep plane facelift under local anesthesia are limited.

The objective of this study is to evaluate the complication rate associated with deep plane facelift surgery performed under local anesthesia and to compare these outcomes with established complication rates reported in the literature.

## METHODS

### Study Design and Patient Population

This was a retrospective clinical study of patients undergoing primary deep plane rhytidectomy under local anesthesia with oral sedation at an office-based surgery facility. Patients with a history of previous facelift surgery were excluded from this analysis given variability in the extent of deep plane dissection. The review of records from September 1, 2023 to June 1, 2025 demonstrated 142 consecutive patients who underwent primary deep plane facelift and necklift under local anesthesia with oral sedation. All surgeries were performed by the author and have at least 90 days of follow-up data. Demographic data collected included patient age, gender, BMI, tobacco use history, medical history, previous facelift surgeries, and current medications. All major intraoperative and postoperative complications were recorded, including hematoma, seroma, skin necrosis, infections, and temporary and permanent facial nerve injuries.

### Patient Selection for Deep Plane Facelift Under Local Anesthesia

Patients seeking out this procedure were medically screened for suitability. Patients on anticoagulation, patients with moderate-to-severe obstructive sleep apnea, and patients with a BMI >40 kg/m^2^ were not offered surgery. Any patients with a history of remote coronary artery disease or stroke required preoperative cardiac clearance. This cohort of patients sought out the performing surgeon for the purpose of having a deep plane facelift under local anesthesia and thus represents emotionally motivated patients. Any patients with apprehension about local anesthesia or patients with a desire to have no awareness of the procedure were recommended to seek out an alternate surgeon offering general anesthesia.

### Local Anesthetic and Oral Sedation

Diazepam was used for oral sedation. Patients took 20 mg of diazepam 30 min before the procedure, after arriving at the facility and having intake and vitals performed. Patients were also instructed to take cephalexin 500 mg or doxycycline 100 mg if allergic to cephalosporins. Patients were also given celecoxib 200 mg and gabapentin 300 mg preoperatively, which were continued twice daily for 1 week postoperatively. The patient was then marked in the seated upright position. The patient was taken to the surgical suite, where they were positioned on the operating table and local anesthesia was administered through a 27 gauge 1.5 inch needle. Total local anesthesia administration for the entire procedure was limited to 7 mg/kg of lidocaine with epinephrine and 3 mg/kg of bupivacaine. Forty milliliters of 0.25% lidocaine with epinephrine 1:400,000 used for local anesthetic of the incision lines in the submental and periauricular areas. Tumescent was injected into all operative areas, from planned incisions extending to limits of dissection, with injections performed in the depth of planned dissection. The tumescent mixture consisted of 10 mL of 2% lidocaine (200 mg lidocaine), 10 mL of 0.5% bupivacaine, 0.8 mL of epinephrine (1 mg/mL), 500 mg of tranexamic acid, and 300 mL of injectable saline. Patients were given a patient-controlled inhalation device of 50% nitrous oxide and 50% oxygen for use as needed during injections (Pro-Nox; Carestream America, Inc., Sanford, FL).

The patient was monitored with continuous pulse oximetry for monitoring oxygen saturation and heart rate. Intermittent blood pressure recordings were obtained by staff with an automatic blood pressure cuff on the upper arm. Between sides on the facelift, when incisions were closed, patients were offered the opportunity for a bathroom break and additional diazepam if needed. Repeat positioning, surgical preparation, and draping were performed after this. There were no significant desaturations or any decreased level of consciousness that was not responsive to verbal or physical stimuli. There were no procedures needing to be aborted or converted, no patients with inadequate tolerance, vasovagal episodes, airway concerns, paradoxical agitation, episodes of hypotension, or other factors disrupting the procedure. The operating suite contained supplementary oxygen, a crash cart, and appropriately trained staff to respond in the case of any emergency.

### Technique

The patient was prepped and draped in standard sterile fashion. A submental incision was made along the internal line of the mandible, and the central skin was undermined past the level of the hyoid and extending laterally to the submandibular glands. No submandibular gland excision or manipulation was performed, given the dangers of encountering uncontrolled bleeding in this area under local anesthesia. The platysma was identified medially, and subplatysmal dissection was carried out laterally along the anterior belly of the digastric muscle to the anterior aspect of the submandibular gland. Lateral fat was debulked with electrocautery, if needed. A 2 cm platysma myotomy was performed at the cervicomental angle bilaterally, and a submental platysmaplasty was performed with interrupted buried sutures of 2-0 Vicryl.

**Figure 1:**
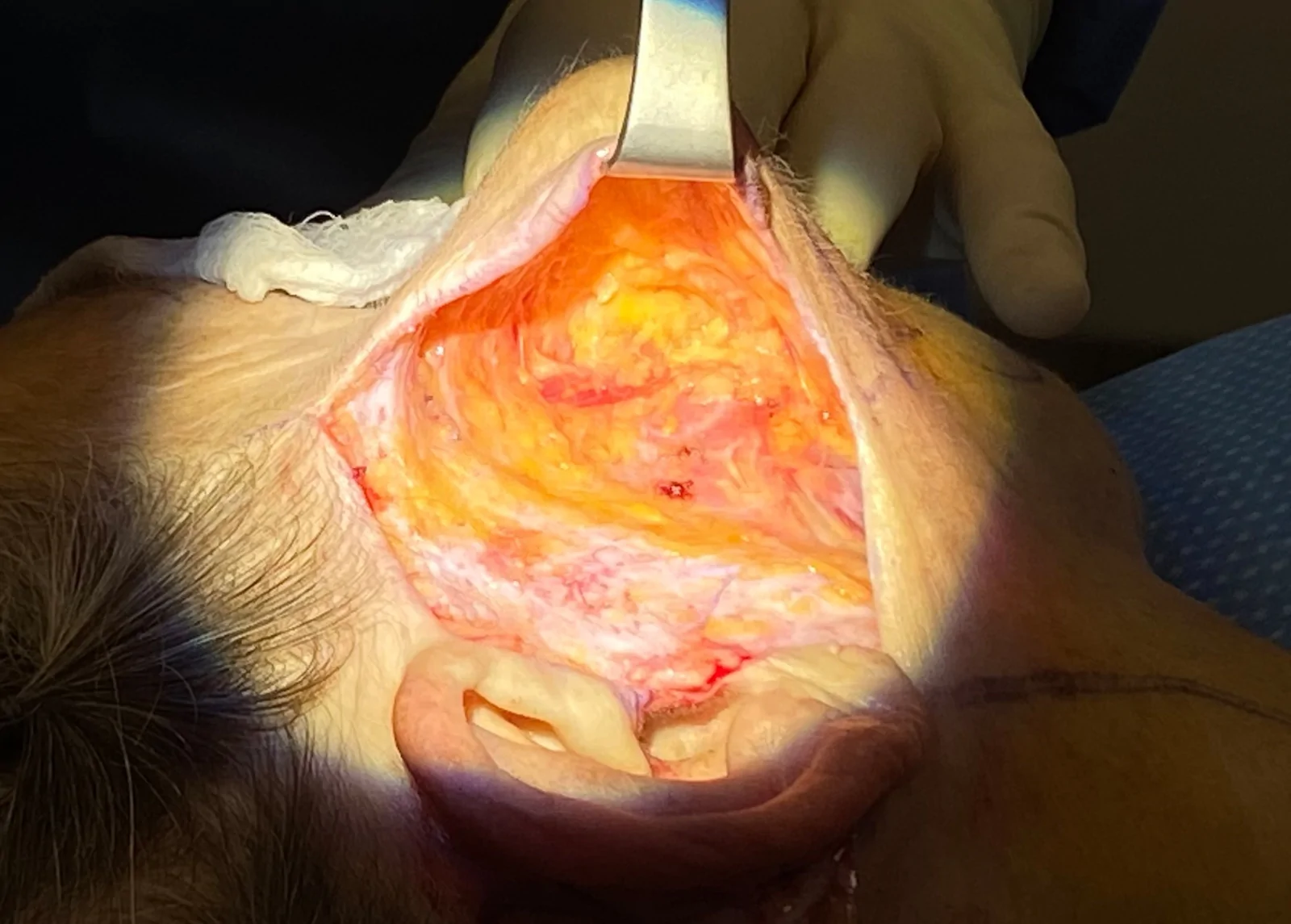
Complete ligamentous release with zygomaticus major muscle fully visualized.

The deep plane facelift was then performed. Standard incisions were made, and skin elevation was carried to the deep plane entry point with sailboat modification.^11^ Incisions were carried into the postauricular sulcus, and a turn was made posteriorly at the junction with the postauricular hairline to allow the incision to lie within the hairline. The skin was elevated posteriorly off the mastoid fascia, and elevation carried over the sternocleidomastoid muscle. The superficial musculoaponeurotic system (SMAS) was sharply entered, and sub-SMAS dissection was carried out anteriorly with a cold knife and blunt dissection. The prezygomatic and premasseteric planes were clearly defined. The zygomatic cutaneous ligament was sharply divided, and the zygomatic major muscle was identified and fully released of overlying attachments (Figure 1). The masseteric ligaments were fully released, and dissection was carried out anteriorly into the loose areolar tissue. The buccal fat was evaluated for excess at this time and was judiciously debulked through the deep plane approach, if needed. The platysma was dissected off of attachments to the tail of the parotid, and subplatysmal dissection was carried out anteriorly to the facial artery. The fully mobilized deep plane composite flap was resuspended in the vertical vector of maximal tissue movement with interrupted buried sutures of 2-0 Vicryl.^2^ The platysma flap was suspended to the lateral mastoid periosteum with sutures of 2-0 Vicryl and 4-0 Nylon. The skin flaps were evaluated, and meticulous hemostasis was achieved with bipolar electrocautery. The skin was redraped and trimmed to ensure no tension on the skin flap. The skin closure was achieved with limited deep dermal sutures of 5-0 Monocryl and a running locking suture of 5-0 fast gut in the preauricular area, 5-0 Vicryl Rapide in the postauricular incisions, and 6-0 Nylon in the submental incision. No drains or hemostatic nets were utilized. Bacitracin was applied to all incision sites, and a compression wrap of fluffed gauze, kerlix, and Coban was placed for 24 h postoperatively, at which point it was removed in clinic and no further compressive garment was used.

The patient was evaluated by the performing surgeon at routine follow-up visits scheduled on postoperative Day 1, 1 week, 2 weeks, 4 weeks, 2 months, 3 months, and 6 months, and clinical examination findings and facial nerve function were documented.

### Analysis

Complication data were compared with the standard in the literature. The aggregate data for deep plane facelifts from the Jacono et al meta-analysis were used to perform odds ratio (OR) analysis.^12^ Analyses were carried out by means of SAS Version 9.4. (SAS Institute, Cary, NC).

## RESULTS

Of the 142 consecutive patients who underwent deep plane facelift and necklift under local anesthesia with oral sedation, there were 137 females (96.5%) and 5 males (3.5%). The average age was 63.5 years with a range of 46 to 80 years old. The average BMI was 25.6 kg/m^2^ (range, 17.3-33.9; Table 1). Of the 142 patients, 37 (26.1%) had an upper blepharoplasty, 19 (13.4%) had a lip lift, and 10 (7.0%) had a facial laser procedure performed concurrent with the deep plane facelift and necklift.

**Table 1:** Demographics and Comorbidities for Study Cohort

	Average	Range
Age (years)	63.5	46-80
BMI (kg/m^2^)	25.6	17.3-33.9

	Number in sample	% of sample
Sex		
Female	137	96.5
Male	5	3.5
Comorbidities		
Hypertension	30	21.1
Diabetes	16	11.3
Thyroid disease	27	19.0
History of tobacco use within the past 3 years	12	8.5
Alcohol intake, reported >1 drink/day	17	12.0

Existence of comorbidities was also evaluated (Table 1). Diabetes mellitus was present in 16 patients (11.3%), hypertension was present in 30 (21.1%), and thyroid disease was present in 27 patients (19.0%). There were 17 patients (12.0%) who reported consuming more than 1 alcoholic beverage per day, although all were requested to stop drinking alcohol 2 weeks before surgery. There were 12 patients (8.5%) with a history of smoking cigarettes within the past 3 years, although all were instructed to stop smoking 6 weeks before surgery at a minimum.

Complications within 90 days of surgery were recorded. There were 5 seromas (3.52%) that resolved with needle aspiration. There were zero major hematomas and 1 minor hematoma, which resolved with needle aspiration (0.70%). There were zero episodes of skin necrosis. There were 2 episodes (1.4%) of local infection characterized by suture redness, which resolved with oral antibiotics. There were zero occurrences of temporary or permanent facial nerve injury. There were no patients requiring hospitalization, and no episodes of deep vein thrombosis or pulmonary embolism were noted within the 90 day postoperative period.

OR analysis was performed to compare complication rates with established complication rates for deep plane facelifts in the literature (Table 2).^12^ The 3.52% rate of seroma in this study was not significantly different than the aggregate 4.0% rate of seroma from the meta-analysis (OR = 0.88, *P* = .82, 95% CI, 0.28-2.74). There was similarly no statistically significant difference in the rate of minor hematoma (0.70% vs 2.81%, OR = 0.25, *P* = .17, 95% CI, 0.03-1.78), major hematoma (0.0% vs 1.22%, OR = 0.28, *P* = .37, 95% CI, 0.02-4.58), skin necrosis (0% vs 0.49%, OR = 0.64, *P* = .76, 95% CI, 0.03-11.19), and infection (1.40% vs 0.73%, OR = 1.94, *P* = .38, 95% CI, 0.44-8.57). There was no significant difference in temporary facial nerve injury (0% vs 0.69%, OR = 0.48, *P* = .62, 95% CI, 0.03-8.26). There were no episodes of permanent facial nerve injury noted in either this study or the aggregate data from the meta-analysis.

**Table 2:** Comparison of Complications Rates of Deep Plane Facelift Under Local Anesthesia With Aggregate Data for Deep Plane Facelift Under General Anesthesia or Monitored Sedation

	Local only—deep plane facelift, %	Deep plane facelift (aggregate, Jacono et al 2019), %	Odds ratio
Seroma	3.52	4.0	0.88 (*P* = .82) 95% CI = 0.28 to 2.74
Hematoma (minor)	0.70	2.81	0.25 (*P* = .17) 95% CI = 0.03 to 1.78
Hematoma (major)	0	1.22	0.28 (*P* = .37) 95% CI = 0.02 to 4.58
Skin necrosis	0	0.49	0.64 (*P* = .76) 95% CI = 0.03 to 11.91
Infection	1.40	0.73	1.94 (*P* = .38) 95% CI = 0.44 to 8.57
Temporary facial nerve injury	0	0.69	0.48 (*P* = .62) 95% CI = 0.03 to 8.26
Permanent facial nerve injury	0	0	None reported in either dataset

## DISCUSSION

**Figure 2:**
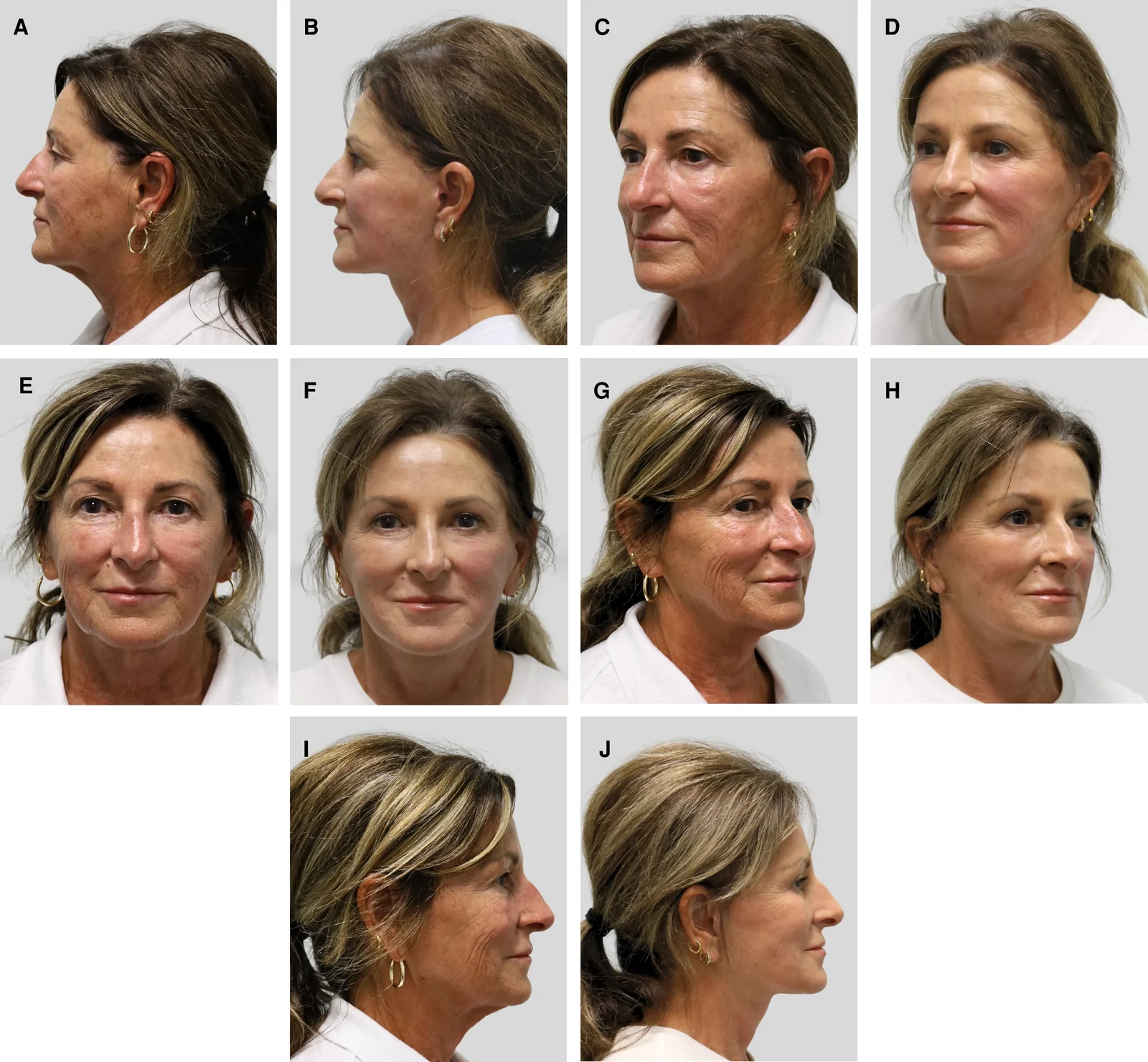
Patient example, a 68-year-old female (A, C, E, G, I) preoperative and (B, D, F, H, J) 6 months post deep plane facelift and necklift with upper blepharoplasty and HALO hybrid fractional laser performed under local anesthesia with oral sedation.

The present study demonstrates that deep plane facelift can be performed safely under local anesthesia with oral sedation in this cohort, with complication rates no higher than published benchmarks for deep plane facelifts performed under general anesthesia or IV sedation. Importantly, the deep plane facelift technique utilized in this series involved full release of the zygomatic cutaneous ligament and complete exposure of the zygomaticus major muscle. Historically, facelifts performed under local anesthesia have been associated with limited dissection techniques. The findings of this study suggest that a deep plane facelift and necklift with complete ligamentous release can be performed safely under local anesthesia (Figure 2).

Concerns regarding hematoma formation with facelift surgery under local anesthesia have been raised in previous literature. One of the largest series examining facelifts under local anesthesia reported a hematoma rate of 13.2%.^9^ In contrast, the current study did not demonstrate any increased risk of hematoma and actually found an overall lower rate, which did not reach statistical significance (0.70% vs 2.81%, *P* = .17). This difference from the Frojo et al paper is most likely attributable to differences in surgical technique rather than anesthetic method. All procedures in this series were comprehensive deep plane facelifts as opposed to a variety of SMAS and high SMAS procedures in the Frojo paper. The tumescent mixture used in this paper also differed in the addition of low-concentration transexamic acid, which has been shown to improve intraoperative hemostasis and may have led to decreased postoperative bleeding.^13^

Another commonly cited concern with facelifts under local anesthesia is lidocaine toxicity. Special attention was given to ensure that total lidocaine administration remained below the accepted limit of 7 mg/kg for injected lidocaine with epinephrine, despite most of the local anesthetic being delivered in tumescent form. Similarly, the bupivacaine dosage was kept below the injection limit of 3 mg/kg. Although safe lidocaine dosing in facelift surgery has been demonstrated in tumescent lidocaine concentrations of 21 mg/kg, this conservative approach was taken to maximize patient safety while still ensuring patient comfort.^14^ No episodes of neurologic symptoms or signs of toxicity were observed in this study. It is particularly important to consider lidocaine dosing in a population of older individuals with increased rates of medical comorbidities, as they may have a reduced lidocaine metabolism and a lower tolerated maximum dosage. Limiting the lidocaine in the tumescent anesthetic also gives additional lidocaine allowance for other facial procedures performed concurrently with the facelift.

In discussing the safety of deep plane facelift under local anesthesia, it is important to note some details of the technique used in this study. Submandibular gland excision was not performed in this series of patients because there is the potential to get into a situation of uncontrolled bleeding requiring extensive cautery and manipulation that would not be well tolerated by an awake patient. It is the author's opinion that submandibular gland excision should not be performed without an IV in place and a plan to administer IV sedation if needed.

This study is not without limitations. The retrospective design introduces inherent biases, and the quality of data collection and documentation limited the ability to evaluate some aspects of the postoperative course and outcomes. Although complication rates were compared with aggregate data from high-level meta-analyses, a matched control group of patients undergoing deep plane facelift under general anesthesia or IV sedation would strengthen the comparison and represents an opportunity for future research. Additionally, this study lacks patient-reported measures, including intraoperative and postoperative experience and outcomes, which will be collected prospectively in a future study.

## CONCLUSIONS

In a selected cohort treated by an experienced single surgeon in an equipped office-based facility, deep plane facelift and necklift under local anesthesia demonstrated low 90 day complication rates. These findings support local anesthesia as a viable and safe alternative for appropriately selected patients undergoing comprehensive deep plane facial rejuvenation.
